# Spin Hall-induced auto-oscillations in ultrathin YIG grown on Pt

**DOI:** 10.1038/s41598-018-19606-5

**Published:** 2018-01-19

**Authors:** M. Evelt, C. Safranski, Mohammed Aldosary, V. E. Demidov, I. Barsukov, A. P. Nosov, A. B. Rinkevich, K. Sobotkiewich, Xiaoqin Li, Jing Shi, I. N. Krivorotov, S. O. Demokritov

**Affiliations:** 1 Institute for Applied Physics and Center for Nanotechnology, University of Muenster, Corrensstrasse 2–4, 48149 Muenster, Germany; 2 Department of Physics and Astronomy, University of California, Irvine, CA 92697 USA; 3 Department of Physics and Astronomy, University of California, Riverside, CA 92521 USA; 4 Institute of Metal Physics, Ural Division of RAS, Ekaterinburg, 620108 Russia; 5 Department of Physics, Center for Complex Quantum Systems, The University of Texas at Austin, Austin, Texas 78712 USA

## Abstract

We experimentally study nanowire-shaped spin-Hall nano-oscillators based on nanometer-thick epitaxial films of Yttrium Iron Garnet grown on top of a layer of Pt. We show that, although these films are characterized by significantly larger magnetic damping in comparison with the films grown directly on Gadolinium Gallium Garnet, they allow one to achieve spin current-driven auto-oscillations at comparable current densities, which can be an indication of the better transparency of the interface to the spin current. These observations suggest a route for improvement of the flexibility of insulator-based spintronic devices and their compatibility with semiconductor technology.

## Introduction

Spintronic devices driven by pure spin current have recently become a subject of intense research^1–14^ owing to their flexible layout not restricted by the requirement of electrical current flow through the active magnetic layer. This advantage is particularly important for the possibility to implement spintronic devices based on insulating magnetic materials, such as Yttrium Iron Garnet (YIG), known for its unmatched small magnetic damping. Additionally, the utilization of magnetic insulators eliminates the shunting of the driving electric current through the active magnetic layer, which is known to be a significant shortcoming of all-metallic spin-current devices^13^. In spite of the significant efforts, the YIG-based spintronics showed a relatively slow progress because of the difficulties associated with the preparation of high-quality nanometer thick YIG films. Only with the recent developments in this field^15–20^, the insulator-based spintronic devices became feasible^8–11^.

The magnetic damping in YIG films is closely related to their structural properties^15–20^. Therefore, high-quality YIG films are usually grown on Gadolinium Gallium Garnet (GGG) substrates providing the precise lattice matching of the two materials, while utilization of other substrates results in a significant increase of the damping constant^21^. This requirement limits the compatibility of YIG with traditional Si-based semiconductor technology. Additionally, it significantly reduces the flexibility of spin current-driven devices utilizing materials with strong spin-orbit interaction such as Pt for generation of spin current by the spin-Hall effect (SHE)^22,23^. Indeed, the requirement that the YIG film has to be grown directly on the substrate forbids fabrication of devices with double-sided injection of the spin current into the magnetic film^24^.

Here we demonstrate experimentally that epitaxial YIG films grown on top of Pt layers can be utilized in SHE-driven spintronic devices. We show that by injecting spin currents with moderate density, one can achieve an efficient suppression or enhancement of magnetic fluctuations, complete compensation of the natural damping, and observe a transition to the magnetization auto-oscillation regime. Surprisingly, in spite of the relatively large damping in YIG grown on Pt, the density of the electrical current necessary for the auto-oscillations onset is very close to that previously reported for high-quality YIG films grown directly on GGG. This observation suggests that the transparency of the YIG/Pt interface to the spin current^25^ is likely improved by growing YIG on Pt.

## Results

### Test devices

**Figure 1:**
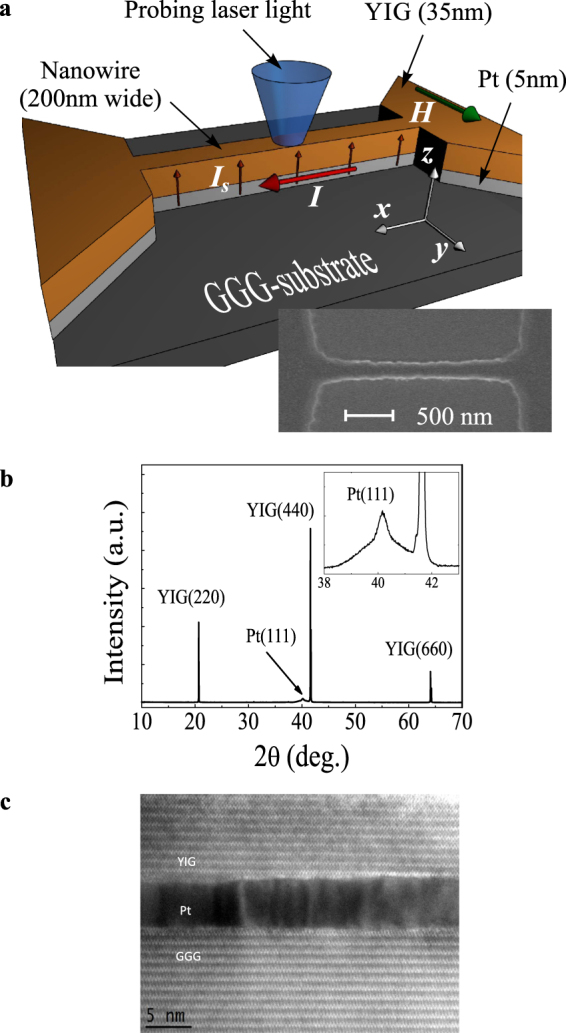
Test devices. (**a**) Schematic of the experiment. Inset shows the SEM image of the device. (**b**) XRD of YIG film grown on Pt/GGG. Inset: Pt 111 peak. (**c**) HRTEM image of YIG/Pt/GGG multilayer.

The schematic of the experiment is shown in Fig. 1a. The studied devices are patterned in a shape of 200 nm wide and 2 μm long nanowires from an epitaxial YIG(35 nm)/Pt(5 nm) bilayer grown on top of a GGG(110) substrate. Figure 1b and c show the results of the structural characterization of YIG/Pt/GGG. The X-ray diffraction (XRD) measurements (Fig. 1b) reveal three main Bragg peaks of YIG and GGG: 220, 440, and 660, suggesting the (110) orientation of YIG and GGG. A small peak at 2*θ*≈40.15° (inset in Fig. 1b) can be identified as the 111 peak of the Pt layer suggesting its (111) texture. The high-resolution transmission electron microscopy (HRTEM) in real space (Fig. 1b) confirms sharp and clean Pt/YIG and GGG/Pt interfaces. The (110) atomic planes of YIG and GGG are parallel to each other and show closely matched inter-planar spacing. This indicates that the crystal orientation of YIG is locked to that of the substrate through single-crystal Pt. Since GGG possesses the same crystal symmetry and nearly the same lattice constant (mismatch ~ 0.056%) as YIG, it is natural to choose GGG as a material for substrate.

The operation of the test device is based on the SHE, which converts the electrical current *I* flowing in the plane of the Pt layer into the out-of-plane spin current *I*_S_. The spin current is injected into the YIG layer through the YIG/Pt interface. It exerts a spin-transfer torque (STT)^26,27^ onto the magnetization in YIG resulting, depending on the direction of *I*, in an increase or decrease of the effective magnetic damping^28^ and in a variation of the intensity of magnetic fluctuations^29^. The device is magnetized by the in-plane static magnetic field *H* applied perpendicular to the axis of the nanowire providing the maximum efficiency of the SHE-induced STT.

### Magnetooptical measurements

We study the effects of the spin current on the dynamic magnetization in YIG by using micro-focus Brillouin light scattering (BLS) spectroscopy^30^. We focus the probing laser light with the wavelength of 473 nm into a diffraction-limited spot on the surface of the nanowire (Fig. 1a) and analyze the light inelastically scattered from magnetic excitations. The resulting signal – the BLS intensity – is proportional to the intensity of magnetic oscillations at the given frequency.

**Figure 2:**
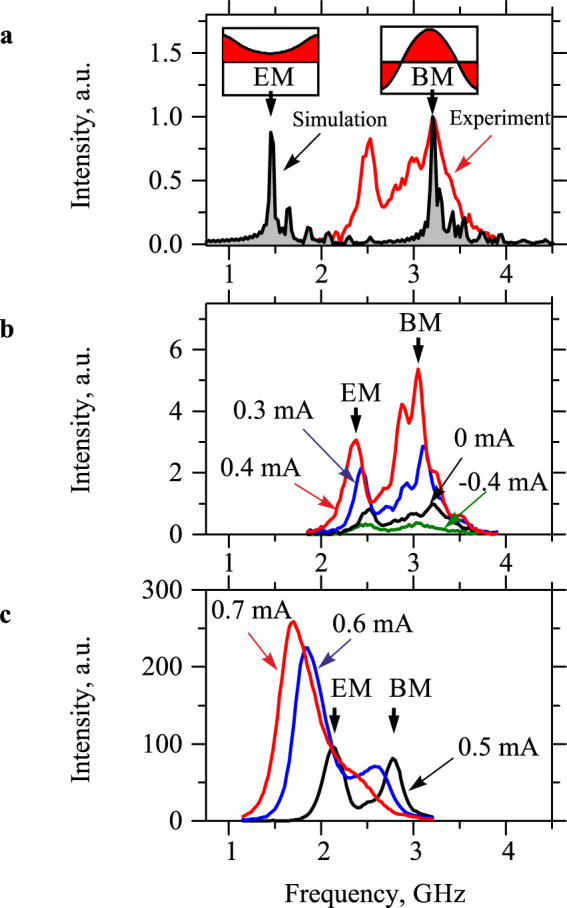
Spectral characteristics of magnetic oscillations. (**a**) Experimental spectrum of magnetic oscillations in the YIG nanowire recorded by BLS at *I* = 0 and the spectrum of magnetic modes obtained for the experimental nanowire geometry from the micromagnetic simulations. Insets show the calculated profiles of the dynamic magnetization across the width of the nanowire for the edge mode (EM) and the bulk mode (BM). (**b)** and (**c**) BLS spectra recorded at different currents in the Pt layer, as labelled. Vertical arrows mark the positions of the peaks corresponding to the edge and the bulk mode at *I* = 0.4 and 0.5 mA in **b** and **c**, respectively. The data were obtained at *H* = 0.5 kOe.

Due to the high sensitivity of the technique, one can detect magnetic oscillations existing in YIG due to the thermal fluctuations at *I* = 0, which enables easy characterization of the initial state of the system before the injection of the spin current. Figure 2a shows the BLS spectrum of magnetic oscillations in the YIG nanowire recorded for *I* = 0 and *H* = 0.5 kOe, together with the spectrum of magnetic modes obtained for the experimental nanowire geometry from the micromagnetic simulations (shadowed) using the MUMAX3 software^31^. The latter was calculated using the independently measured saturation magnetization of YIG 4π*M*_0_ = 1.6 kG. The Gilbert damping constant was chosen to be artificially small (α = 5×10^−4^) to enable clear identification of the spectral peaks corresponding to different modes. The obtained spectrum shows two intense peaks marked in Fig. 2a by the vertical arrows. These peaks correspond to the two fundamental dynamic modes of the system – the edge mode (EM) and the bulk mode (BM) – characterized by different distributions of the dynamic magnetization in the direction perpendicular to the axis of the nanowire, as shown in the insets in Fig. 2a. Additional small-amplitude peaks correspond to the higher-order edge and bulk modes quantized in the direction along the nanowire^5^. Comparison of the simulated spectrum with that obtained by BLS shows a perfect agreement in the frequency of BM, while the experimentally obtained frequency of EM 2.5 GHz is higher than that observed in simulations. We emphasize that this result is in a good agreement with previous studies^10,32^. In particular, in ref.^32^, it was shown that, in magnetic nanowires, the experimentally observed frequencies of the edge modes are always higher than those obtained from micromagnetic simulations. This frequency mismatch was attributed to an extrinsic pinning of the magnetization at the edges of the nanowire due to the joint action of the edge roughness, edge dilution effects, the non-zero surface anisotropy, etc. Recently similar behaviors have also been reported for YIG nanowires with dimensions close to those used in this work^10^. We emphasize that, in spite of the observed mismatch, based on the results of micromagnetic simulations, one can clearly identify the experimental peak at 2.5 GHz as the EM peak.

Figure 2b and c illustrate modifications of the BLS spectra under the influence of the spin current. With the increase of *I* > 0, the intensities of both spectral peaks increase reflecting the current-induced enhancement of magnetic fluctuations^29^. The increase of the amplitudes of fluctuations with current results in the reduction of the effective static magnetization leading to the nonlinear redshift^33,34^ of the frequencies of the modes. Additional contribution to the frequency shift comes from the reduction of the effective static magnetization due to the Joule heating of the YIG film by the current flowing in the Pt layer. In particular, it results in a reduction of the frequencies of the modes at *I* < 0, where the long-wavelength magnetic fluctuations are suppressed by the spin current (see spectrum for *I* = −0.4 mA in Fig. 2b).

Note that, at *I* > 0, the intensity of the EM peak, which is initially smaller than that of the BM peak (Fig. 2a), grows noticeably faster. The intensities of the peaks become approximately equal at *I* = 0.5 mA and the EM peak starts to strongly dominate the BM peak at larger currents. These behaviors are in agreement with the concept of the influence of the spin current on the chemical potential of magnons^35^, which generally predicts stronger influence of the spin current on the magnetic modes with lower frequencies.

**Figure 3:**
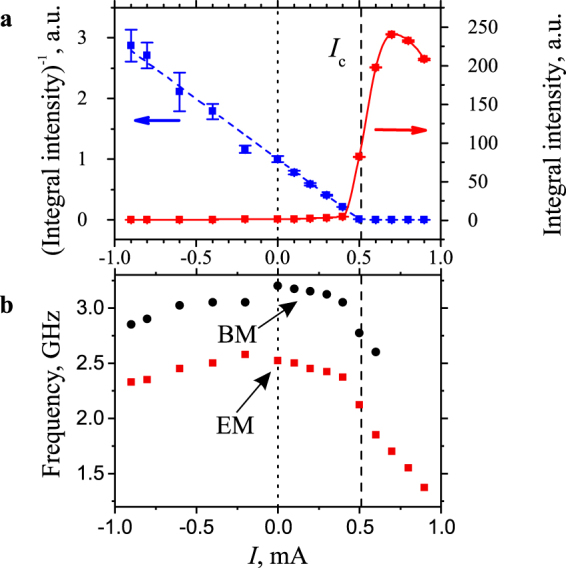
Current dependences. (**a**) Current dependence of the normalized BLS intensity integrated over the spectrum and of its inverse value. Symbols – experimental data, solid curve – guide for the eye, dashed line – linear fit of the experimental data for the inverse integral intensity at *I* < 0.4 mA. (**b**) Current dependences of the frequencies of the edge mode (EM) and the bulk mode (BM). *I*_C_ marks the critical current corresponding to the onset of magnetization auto-oscillations. The data were obtained at *H* = 0.5 kOe.

Figure 3a shows the current dependence of the BLS intensity integrated over the spectrum, which reflects the total energy stored in magnetic modes accessible by BLS, and of its inverse value. As seen from these data, the enhancement of magnetic fluctuations is only observed for positive *I*, which is in agreement with the symmetry of SHE. The integral intensity grows slowly at *I* < 0.4 mA and then shows an abrupt increase at *I*≈0.5 mA followed by a saturation at *I* > 0.7 mA. These behaviors suggest that, at *I* ≈ 0.5 mA, the spin current completely compensates the intrinsic magnetic damping in YIG resulting in the onset of magnetization auto-oscillations. In agreement with the general theory of devices driven by STT^36^, the inverse of the integral intensity shows a linear dependence at *I* < 0.5 mA typical for the effect of STT on magnetic fluctuations. We emphasize that, at *I* < 0, the integral intensity decreases with respect to that at *I* = 0 by about a factor of three. This corresponds to the current-induced reduction of the thermal magnetic noise – a phenomenon, which has been previously observed for SHE systems based on metallic magnetic films^29^. We also note that the precisely linear current dependence of the inverse integral intensity clearly shows that the observed behaviors are solely caused by SHE, while the spin Seebeck effect^37^ important in Pt/YIG/GGG devices^10,11^ plays a negligible role in the YIG/Pt/GGG devices studied here. The YIG and Pt layer sequence matters because the Pt/YIG/GGG configuration maximizes current-induced temperature gradient across the YIG film by sandwiching it between the Pt ohmic heat source and the GGG substrate heat sink^10^.

The linear current dependence of the inverse integral intensity (Fig. 3a) allows the precise determination of the auto-oscillation onset current. According to the theory^36^, this dependence crosses zero at the current, corresponding to the complete compensation of the natural magnetic damping in YIG by the current-induced STT. By fitting the experimental data for the inverse integral intensity by a linear function and finding its zero intercept, we determine the auto-oscillation onset current *I*_C_ = 0.51 mA. At this current, both the edge and the bulk mode switch to the auto-oscillation regime resulting in multi-mode auto-oscillations, similarly to all-metallic nanowire SHE oscillators^5^. The additional separate analysis of the current-dependent intensities of the edge and the bulk mode shows that *I*_C_ is approximately the same for both modes.

Figure 3b shows the current dependences of the frequencies of the spectral peaks corresponding to the bulk and the edge mode. At |*I*| < *I*_C_, the frequencies of both modes reduce only slightly. The frequency shift is approximately the same for positive and negative currents indicating that the Joule heating of the YIG layer by the current plays the dominant role at small *I*. As the current approaches *I*_C_, the frequencies of both modes start to decrease quickly. This decrease is well correlated with the abrupt increase of the BLS intensity (Fig. 3a) confirming the dominating contribution to the frequency shift of the large intensity of magnetic oscillations excited by the spin current.

**Figure 4:**
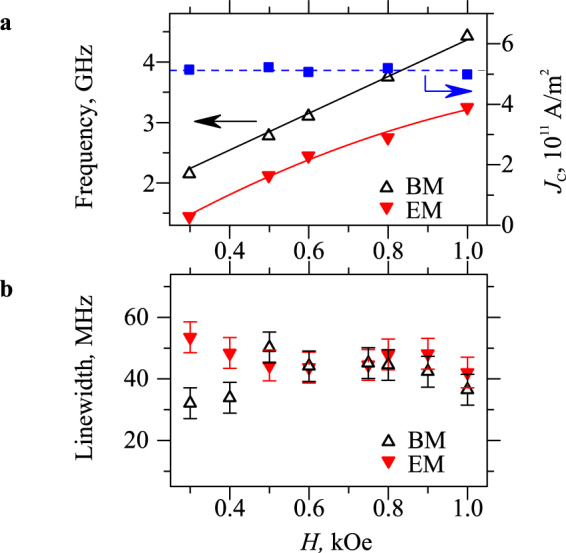
Static-field dependences. (**a**) Static-field dependencies of the density of the auto-oscillation onset current *J*_C_ (squares) and of the frequencies of EM and BM at the onset of auto-oscillations (triangles). Symbols – experimental data, solid curves – guides for the eye, dashed line – mean value of *J*_C_. (**b**) Static-field dependencies of the spectral linewidth of EM and BM obtained from FMR-BLS measurements at *I* = 0.

Behaviors similar to those described above were observed in a broad range of the static magnetic field *H* = 0.3–1.0 kOe. We show in Fig. 4a the obtained field dependencies of the density of the auto-oscillation onset current *J*_C_ (squares) and of the frequencies of EM and BM at the onset of auto-oscillations (triangles). As seen from these data, within the studied range of *H*, the frequencies of the modes vary by more than a factor of two, while *J*_C_ ≈ 5×10^11^ A/m^2^ remains nearly constant.

## Discussion

The observed independence of *J*_C_ from the static magnetic field is surprising, since the density of the onset current is known to be proportional to the relaxation rate Γ of the dynamic modes^36,38^, which generally increases with the increase of the mode frequency. In the approximation of an extended magnetic film^36^, the relaxation rate $\Gamma =\alpha \gamma (H+2{\pi }{M}_{0})$ is expected to increase by about 50% as the static field varies from 0.3 to 1.0 kOe, which disagrees with the experimental data for *J*_C_ (Fig. 4a). As was shown in ref.^8^, for YIG films with typically small Gilbert damping constant α, the additional contribution to the magnetic relaxation due to the inhomogeneous broadening effects can be significant. Taking into account that this contribution is independent of the static field (frequency), one can suggest that it fully dominates the total relaxation rate in the studied structures resulting in a field-independent onset current.

To prove this assumption we perform high-resolution ferromagnetic-resonance (FMR)-BLS measurements of the spectral linewidth of the observed magnetic modes. We excite the magnetic dynamics in the YIG nanowire by the microwave-frequency magnetic field produced by an ac current in the Pt layer and detect the magnetization response by BLS with *H* rotated by 5° from the direction perpendicular to the nanowire axis (see ref.^39^ for details). The obtained results (Fig. 4b) show that, within the measurement accuracy, the linewidths of the EM and BM are approximately equal and remain constant over the range *H* = 0.3–1.0 kOe. Since the linewidth directly characterizes the damping rate of the dynamic magnetization, we conclude that, in the YIG films grown on Pt, the field-dependent Gilbert-like contribution to the damping is much smaller compared to the field-independent contribution associated with the inhomogeneous broadening effects.

Finally, we quantitatively compare the characteristics of our devices with those obtained for devices based on YIG grown on GGG. The minimum onset current density 4×10^11^ A/m^2^ observed in the latter case^8^ is smaller by only 20% than that in our devices based on YIG grown on Pt. However, the typical linewidth in the frequency range of 2–4 GHz reported in ref.^8^ is by about a factor of two smaller than that in our devices. This suggests, that the significantly larger damping in YIG grown on Pt is compensated by the higher efficiency of SHE, which can be associated with the better transparency of the YIG/Pt interface to the spin current.

In conclusion, we have demonstrated that efficient spin current-driven devices can be made from YIG films grown on Pt, where the auto-oscillation characteristics are not adversely affected by the unavoidably increased magnetic damping. Our observations suggest a route for implementation of highly flexible insulator-based spintronic devices, where both interfaces of the magnetic-insulator film are available for spin-current injection. Our results should stimulate further development of spintronic devices based on magnetic insulators.

## Methods

### Sample fabrication

The details of the growth of the YIG/Pt bilayer as well as its structural and static magnetic characterization can be found in ref.^20^. The patterning of the bilayer is performed by defining a negative resist etching mask in the shape of a nanowire with two contact pads via a single e-beam lithography step. The mask pattern is then transferred to the film via ion mill etching, and the remaining resist is removed by oxygen plasma cleaning.

### Magneto-optical measurements

Micro-focus BLS measurements were performed by focusing light produced by a continuous-wave single-frequency laser operating at the wavelength of 473 nm into a diffraction-limited spot. The light scattered from magnetic oscillations was analyzed by a six-pass Fabry-Perot interferometer TFP-1 (JRS Scientific Instruments, Switzerland) to obtain information about the BLS intensity proportional to the square of the amplitude of the dynamic magnetization at the location of the probing spot. All measurements were performed at room temperature.

### Simulations

The micromagnetic simulations were performed by using the software package MuMax3 (ref.^31^). The computational domain with dimensions of 2×0.2×0.035 µm³ was discretized into 20×4×35 nm³ cells. The FMR mode was excited by a 15 ps wide sinc pulse of the out-of-plane magnetic field. The spatial map of the dynamic magnetization was reconstructed based on the Fourier analysis of the dynamic response of the magnetization. The standard value for the exchange stiffness of 3.66×10^−12^ J/m was used, while the value of the saturation magnetization 4π*M*_0_ = 1.6 kG was determined from the BLS spectra of thermally excited magnons at *I* = 0 mA. The Gilbert damping constant of 5×10^−4^ was chosen artificially low to allow for a low pulse width and therefore enable a high resolution of the excited modes.
